# Examining the Potential of a Random Forest Derived Cloud Mask from GOES-R Satellites to Improve Solar Irradiance Forecasting

**DOI:** 10.3390/en13071671

**Published:** 2020-04-03

**Authors:** Tyler McCandless, Pedro Angel Jiménez

**Affiliations:** National Center for Atmospheric Research (NCAR), Boulder, CO 80305, USA

**Keywords:** solar power forecasting, machine learning, artificial intelligence, random forests, supervised learning, remote sensing

## Abstract

In order for numerical weather prediction (NWP) models to correctly predict solar irradiance reaching the earth’s surface for more accurate solar power forecasting, it is important to initialize the NWP model with accurate cloud information. Knowing where the clouds are located is the first step. Using data from geostationary satellites is an attractive possibility given the low latencies and high spatio-temporal resolution provided nowadays. Here, we explore the potential of utilizing the random forest machine learning method to generate the cloud mask from GOES-16 radiances. We first perform a predictor selection process to determine the optimal predictor set for the random forest predictions of the horizontal cloud fraction and then determine the appropriate threshold to generate the cloud mask prediction. The results show that the random forest method performs as well as the GOES-16 level 2 clear sky mask product with the ability to customize the threshold for under or over predicting cloud cover. Further developments to enhance the cloud mask estimations for improved short-term solar irradiance and power forecasting with the MAD-WRF NWP model are discussed.

## 1. Introduction

Numerical weather prediction (NWP) models require an accurate cloud initialization to provide reliable solar irradiance forecasting. This is especially the case for short term forecasts or nowcasts where the forecast window is only a few hours long [1,2]. Given the emphasis placed in the short term, nowcasting systems require low latency in any potential data source used to initialize the clouds in the model.

Data from the current generation of sensors on geostationary satellites are well suited for nowcasting applications [3]. These satellites provide high-spatio temporal resolution sampling in the visible and infrared with similar spectral resolution as polar orbiting satellites. For example, the current generation of Geostationary Operational Environmental Satellites (GOES) satellites, GOES-R series, sample a total of 16 bands every 10 min over the full disk of the Earth [4]. Having good spectral coverage is essential to determine cloud properties. The data is available shortly after the samples are processed, which provides around 1 min latencies to determine cloud properties for NWP initialization.

The first step in the initialization is knowing where the clouds are located. This cloud mask, or clear sky mask, can then be inserted in the model initialization [5]. This is the approach that we are using to initialize clouds in the MAD-WRF nowcasting system. In MAD-WRF we are blending two nowcasting systems to provide an improved end-to-end model for solar irradiance nowcasting. The systems being a modified version of the Multi-Sensor Advection Diffusion Nowcast (MADCast, [6]) that advects and diffuses the clouds as tracers using the Weather Research and Forecasting (WRF, [7]) model, and a NWP model tailored for solar energy applications, WRF-Solar [8]. Both models, MADCast and WRF-Solar, and therefore MAD-WRF, are tightly coupled with a cloud initialization system. The initialization of the cloud mask combines data from the GOES-R series satellites and meteorological variables available at the initialization time using a machine learning model based on the random forest (RF) algorithm. This RF approach has the potential to capture non-linear relationships within the satellite and meteorological variables in relation to whether a cloud is present in a given area, which would provide highly advantageous for improving short-term solar power forecasts.

Solar radiation is largely affected by feedbacks between atmospheric dynamics and cloud microphysical processes like condensation and evaporation [8], which are represented by NWP models such as MAD-WRF. The initialization of the models with the correct cloud location is essential for accurate predictions. The cloud mask at initial time is the most important cloud characteristic to properly represent since a cloud misplacement typically has a larger impact on the surface irradiance than misrepresentations of other cloud properties such as the hydrometeors content or the radius of the hydrometeors. In addition, a misplacement of clouds can lead to undesired nonlinear effects as a result of cloud microphysics interactions with atmospheric dynamics and thermodynamics and the different physics schemes within MAD-WRF. As a result, by more accurate representations of the location of clouds at the initialization time, MAD-WRF may better capture not only the short-term irradiance but also the cloud field evolution at longer lead times [8]. It has been shown that advances such as these have allowed NWP models to become more accurate in shorter-term forecasts [9,10], which bridges the gap to the benefit of nowcasting methodologies such as machine learning or satellite-based cloud advection techniques [11,12]. A machine learning approach to improve the initialization of clouds would have a significant impact on improving short-term solar irradiance and power forecasts due to the reduction of cloud misplacements at initial time and better representation of cloud microphysics interactions with atmospheric dynamics and the physical processes modeled in NWP.

A novel aspect in our approach is that we do this inside the NWP model, so the clouds are consistent with the model meteorological variables. A summary of previous research in cloud detection has been presented in [13] with many machine learning technologies used from convolution neural networks on images [14], Bayesian classifier [15], decision tree [16], boosting and RF [17] among others. However, these methods did not utilize GOES-R data in the training; they did not directly compare to the GOES Level 2 product for cloud detection, and they did not utilize a WRF specifically tuned to improve solar irradiance forecasting.

The main aim of this work is to explore the potential of utilizing machine learning within an NWP model, MAD-WRF, to improve the state-of-the-art detection of clouds from satellite data in order to improve cloud initialization in short-term solar irradiance forecasts. We focus over a grid covering the Contiguous U.S. (CONUS) at 9 km of grid spacing, which is adequate for nowcasting applications. Meteorological variables available at initialization time and data from a GOES-R satellite, GOES-16, are used as predictors for the RF model. The RF machine learning approach was chosen due to its rule-based technique with decision boundaries well suited for cloud mask prediction while capturing non-linear relationships among the predictors. The RF is an interpretable machine learning technique that can be used to better understand the benefit of GOES-R data and predictability of the cloud mask for improving short-term solar irradiance and power forecasts.

## 2. Materials and Methods

### 2.1. Predictand and Predictor Data

Three different sources have been used to create the predictand and predictor data used in training and testing the machine learning algorithm. The predictand data was generated using cloud retrievals from the Cloud-Aerosol Lidar and Infrared Pathfinder Satellite Observations (CALIPSO, [18]) platform. The predictors were obtained from a geostationary satellite, GOES-16, and from an operational NWP model, the High Resolution Rapid Refresh (HRRR, [19]) model. Data from HRRR is typically used is typically used to initialize NWP predictions over CONUS, the region under consideration. Both predictor datasets are internally archived at NCAR. The predictand and predictor datasets comprise the period from 1 January 2018 to 30 September 2019. The spatio-temporal matching of the datasets has been performed by projecting the data nearest to each hour to a WRF grid that covers CONUS at 9 km of horizontal grid spacing. The following paragraphs provide specific details of this process for each of the datasets.

**Figure 1.**
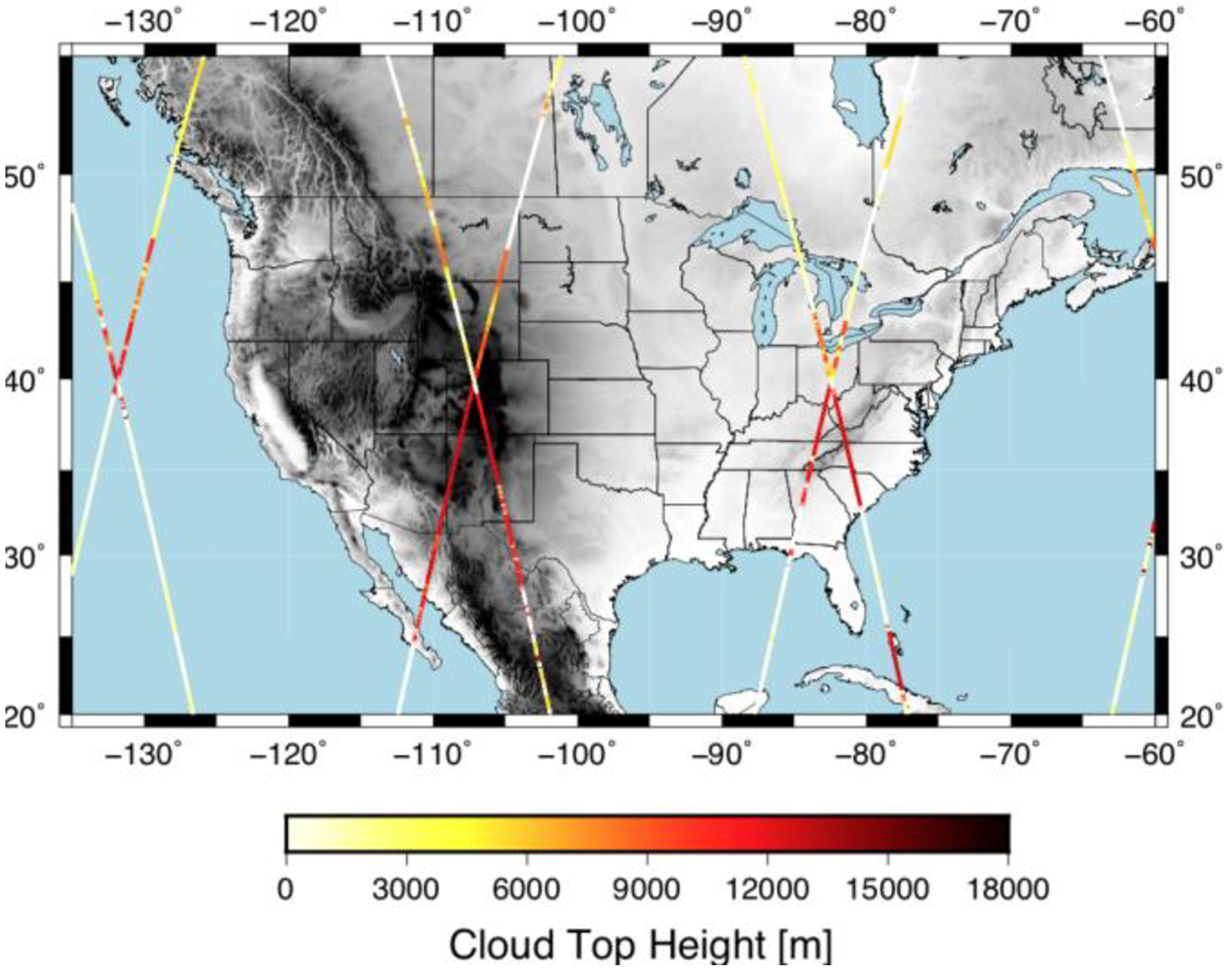
Cloud top height retrievals from CALIPSO during 1 April 2018.

CALIPSO is part of the A-Train constellation [20] that orbits the Earth in a sun-synchronous orbit. In this orbit, there are typically four passes per day over CONUS. This can be appreciated in Figure 1 that shows the cloud top height retrievals from the 1-km Cloud Layer product (ValStage1 v3–40) for 1 April 2018. This data has been used to calculate the horizontal cloud fraction at each of the coincident WRF grid points with at least two retrievals available. The horizontal cloud fraction data is assigned to the nearest hour.

GOES-16 is the geostationary satellite operational on the GOES East position, 75.2 degrees west, since 18 December 2017. GOES-16 is the first of the GOES-R series and provides significant enhancements with respect to the previous GOES satellites. GOES-16 is equipped with the Advanced Baseline Imager (ABI, [4]) and provides images at 0.5, 1, or 2 km, depending on the observation band. The ABI spectral coverage is similar to polar orbiting sensors like the Moderate Resolution Imaging Spectroradiometer (MODIS) or the Visible Infrared Imaging Radiometer Suite (VIIRS). In particular, it provides observations at 16 channels. The first 6 are distributed over the visible and near infrared and the rest over the infrared. The data is available every 10 minutes for the full disk scan. Data from the full disk scans at the 16 GOES-16 channels have been used as predictors. With this aim, we have projected the reflectances (bands 1–6) and brightness temperatures (bands 7–16) to our target grid. The data nearest to the top of each hour was selected if it was within 800 s of the top of the hour.

In addition to the GOES-16 bands, we added as predictors meteorological variables from the HRRR analysis. HRRR is an operational NWP model run by NCEP on an hourly basis covering CONUS at 3 km of grid spacing. The variables selected are the 2-m temperature, skin temperature, snow content, surface albedo, relative humidity at 15, 100, 250, 500, 750 and 1000 m above the ground level, the maximum relative humidity in the vertical column and the maximum relative humidity with respect to ice in the vertical column. These variables were projected to our 9-km grid. The HRRR and GOES-16 variables were complemented with the solar zenith angle to complete the predictor dataset.

The predictand and predictor data were split into training and testing datasets (supplementary materials). The testing dataset contains approximately ⅓ of the records chosen by randomly picking one month of each season (January 2018, April 2018, July 2018, October 2018, February 2019, May 2019 and August 2019) to hold out for independent validation. The training dataset included the remaining data and was further subset with 80% of records remaining in the training dataset and 20% in a validation dataset. The validation dataset was used to find the optimal configuration of the models such as determining the best hyperparameter configuration of the RF model.

### 2.2. Random Forest Algorithm

**Figure 2.**
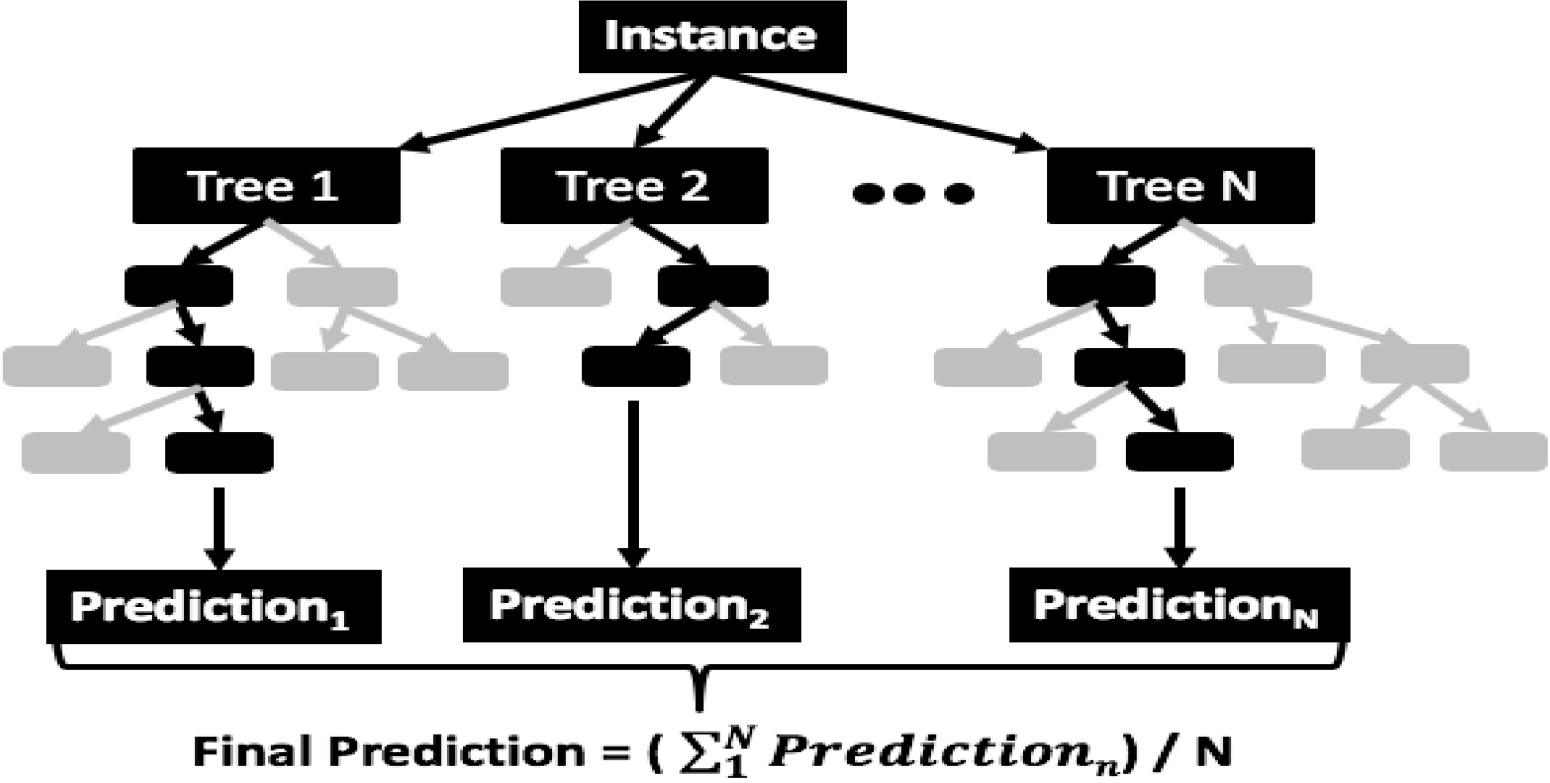
Illustration of the random forest (RF) where the final prediction is the average of the predictions of each tree in the model.

The RF algorithm has grown in popularity in recent years due to its interpretability and its general ability to avoid over-fitting. RFs are a combination of a specified number of decision or regression trees [21]. Regression trees are a rule-based technique used for predicting continuous variables where the nodes are split based on their impurity as measured by the residual sum of squares, which is a measure of how the observations at a given branch fit the model [22]. The regression tree starts with creating a rule to branch the tree based on the highest reduction in variance and proceeds until the final branch has a minimum variance, which is the final leaf node. The final prediction for a continuous variable, such as cloud fraction, is the mean of the instances in the final leaf node for an instance that follows the rules of the branches down to the final leaf. This is illustrated by the green decision nodes in Figure 2 that depicts a random forecast model, which is described in further detail in [23]. In this illustration, the darker boxes indicate how a RF model would make a prediction for a given instance by following the set of rules in each tree and computing the ensemble average of the prediction from each tree in the forest. While the RF performs well for interpolation, it does not extrapolate due to the mean value for the training data being used as the prediction in the final leaves of the trees. Given that we are trying to predict cloud fraction in a range of 0 to 1, the lack of extrapolation is not an issue in this prediction problem. The RF specifically handicaps each tree in the forest with a subset of the available predictors and the available training data; however, by taking the ensemble average of the prediction from each tree in the forest, the model tends to avoid overfitting and thus has a lower error on average than any tree in the forest. One of the benefits of the RF technique is that it is interpretable by examining the rules of the tree with metrics such as predictor importance and partial dependence plots as described in Sections 3 and 4.

## 3. Results

### 3.1. Predictor Selection

The first step in configuring the machine learning model is to determine the optimal predictors into the model. This is important in order to eliminate predictors that have multicollinearity characteristics and eliminate predictors that are irrelevant, thereby improving the model’s accuracy in operational use. In this study, we use multiple methods in order to determine the best predictor set for the machine learning model. First, we use correlation analysis to better understand the correlations among the predictors and with the predictand. Second, we use predictor importance to gain knowledge on the RF’s decisions in splitting the branches in the decision trees. Third, we perform recursive feature elimination (RFE) to determine the predictors that can be removed from the dataset without significant loss of error. Finally, we combine these steps with our physical knowledge of the predictor-predictand relationship to determine our final predictor set.

#### 3.1.1. Correlation Analysis

**Figure 3.**
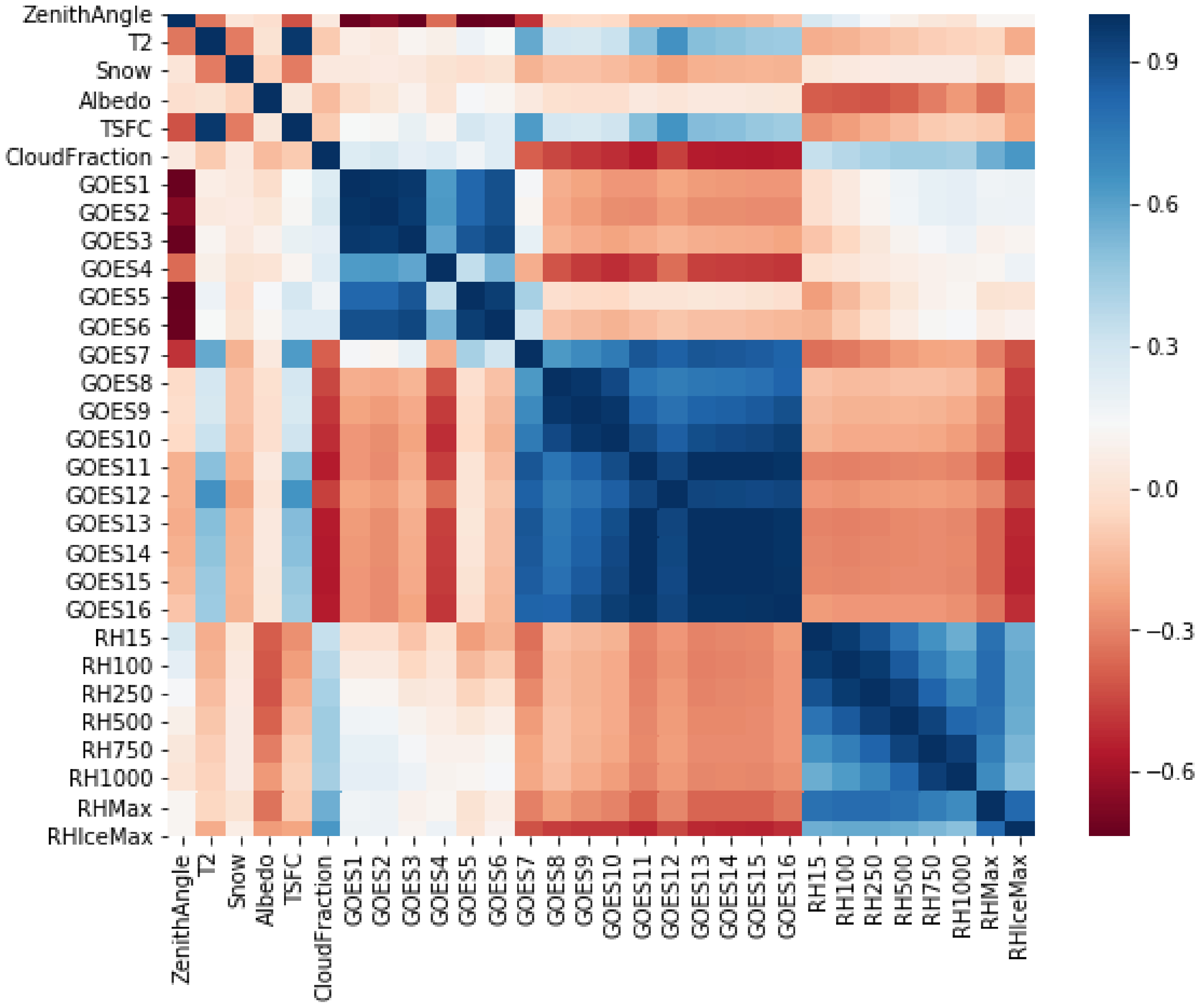
Correlation matrix for all predictors and predictand (Cloud Fraction) indicating there is multicollinearity in the range of Geostationary Operational Environmental Satellites (GOES) 1 through 6, GOES 7 through 16, and all relative humidity (RH) levels.

Correlation analysis is an important first step to better understand the purpose of each of the predictors in the machine learning algorithm. Correlation analysis quantifies the linear relationship between two variables where predictors with high correlation are linearly dependent [24]. Here, we evaluate the correlation matrix to determine which predictors are correlated with each other and the relationship between individual predictors and the predictand (Cloud Fraction). Figure 3 shows the correlations among all the predictors and the predictand. This analysis shows that there is substantial multicollinearity between the GOES predictors as indicated by the dark blue colors in correlations between GOES channels 1–6 and GOES channels 7–16. This makes physical sense as GOES channels 1–6 are the visible and near infrared (IR) channels while 7–16 are the IR channels. Multicollinearity occurs when you can predict one predictor variable as a linearly from other predictor variables; therefore, giving the RF all of these predictors would limit the accuracy of the rule-based technique. This is due to duplicative predictive skill resulting into inconsistent rule-splitting and overfitting of chance relationships in the training data. We will next evaluate predictive importance and recursive feature elimination to determine which of the predictor variables should be removed.

#### 3.1.2. Predictor Importance

**Figure 4.**
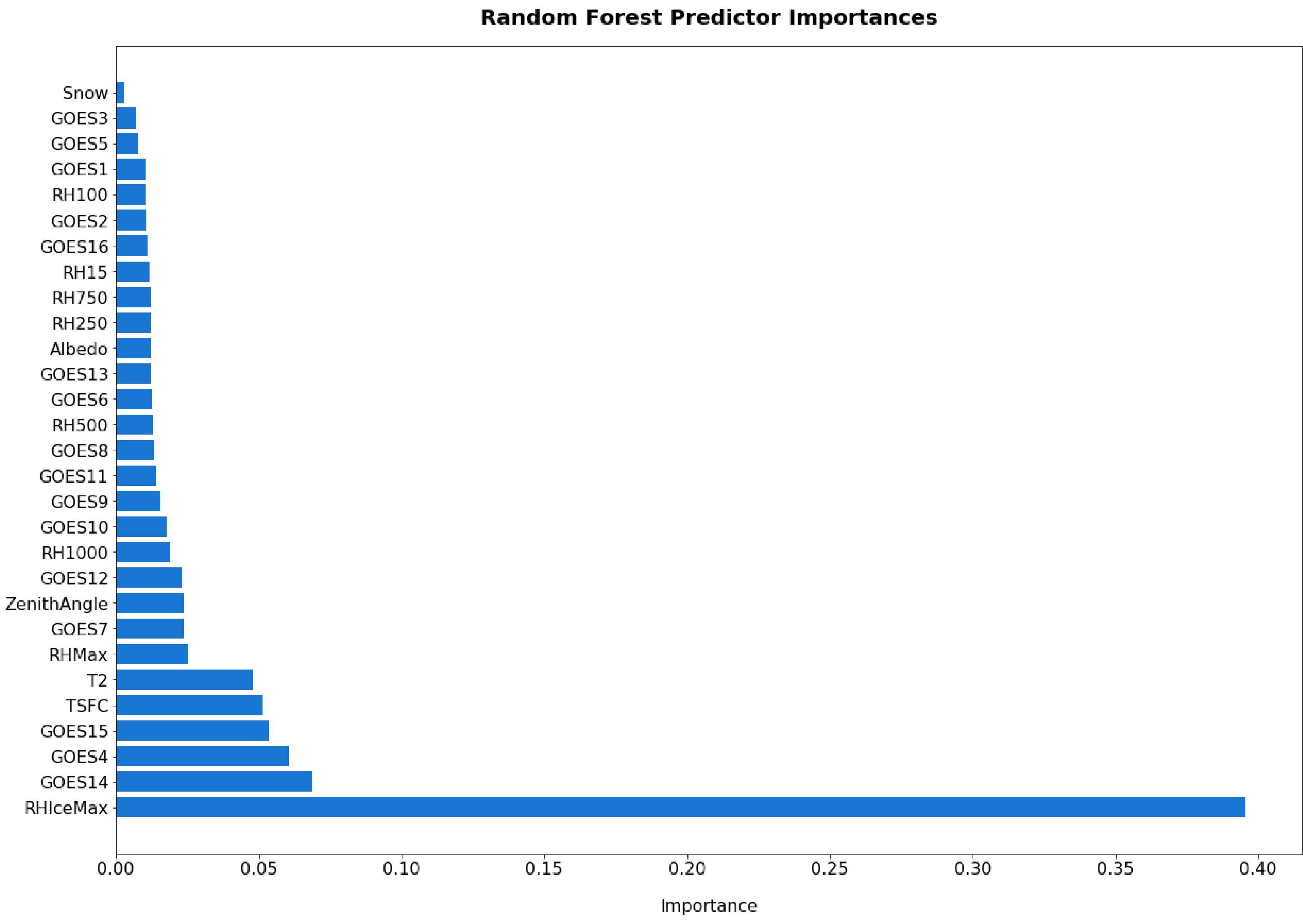
RF predictor importances, which highlight that the maximum relative humidity of the ice cloud layers (RHIceMax) is the most important predictor followed by the GOES channel 14.

In order to gain a better understanding of the value of each of the predictors, we examine the predictor importance for the RF model. The RF model is an ensemble of regression trees where each tree in the forest is given a subset of the available predictors and training data. The RF predictor importance is computed as the average across all trees of how much each predictor contributed to decreasing the weighted variance using the Scikit-Learn feature selection package [25]. This approach does have a tendency to overstate the importance of continuous variables, but provides evidence in the physical interpretation of the predictors in the RF. The predictor importance for the RF is shown in Figure 4, which identifies that the maximum relative humidity with respect to ice in the vertical column, and GOES channels 14, 4 and 15 are the most important predictors. Snow has the least amount of predictive skill; however, that is expected in the model as there are few relative instances of snow in the dataset and it is a categorical variable. We may opt to override the recursive feature elimination of removing snow as a predictor since that snow will cause the satellite reflectances to be in error and affect the predictive error in those relatively few instances.

#### 3.1.3. Recursive Feature Elimination (RFE)

**Figure 5.**
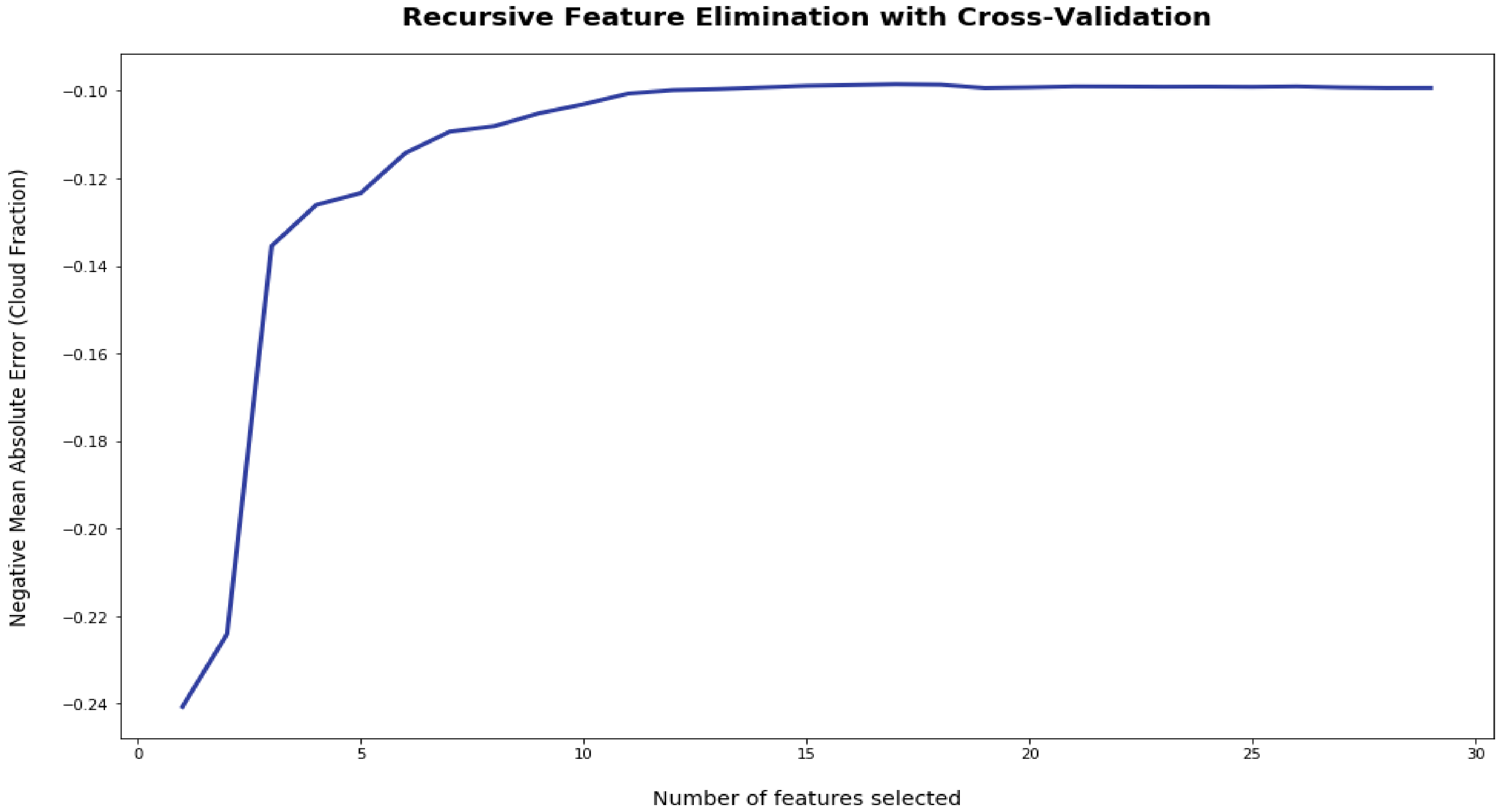
Recursive Feature Elimination that shows the negative mean absolute error as a function of number of features selected. The optimal number of features was determined to be 17 that had the lowest mean absolute error (MAE) (most negative MAE in plot).

Finally, we perform recursive feature elimination (RFE, [26]) that systematically removes the predictor with the lowest importance and repeats the model training and error evaluation. When the RFE begins to show increasing error by removing predictors, that is generally the point at which the predictors removed are not included in the final model since they are not adding value to the predictions; however, we may opt to keep certain variables such as snow due to the fact that we know these are rare instances but have significant predictive information. We utilize python’s Scikit-Learn RFECV package that performs stratified k-fold validation with a mean absolute error (MAE) scoring metric and a k of 3 [25]. These RFE results indicate that 17 predictors are the optimal number; however, it is clear that there is minimal increase in error by including the additional predictors (Figure 5). Seventeen predictors were chosen by the RFE results since it has the lowest statistically significant MAE while minimizing number of predictors. Ultimately from Figure 5, it is clear that the results are quite similar between 13 and 29 predictors. The predictors chosen to be eliminated include ones expected of multicollinearity, including multiple RH levels and GOES channels. The GOES channels chosen to be eliminated include channels 1, 2, 3 and 5, which are in the visible to near-IR spectrum and channels 13 and 16 in the IR spectrum. This is logical as GOES channel 4 and 6 may incorporate the predictive skill of the visible to near IR range from 1 through 6. Similarly, the relative humidity levels chosen to be eliminated are levels at 15, 100, 500 and 750 m, and the relative humidity levels kept in the final predictor set include 250 and 1000 m, maximum in column and maximum with respect to ice. Additionally, albedo and snow were two other predictors that RFE determined should be eliminated. However, as previously stated, we kept snow in the final predictor set due to the physical reasoning and the low number of observations that limited its reduction in variance.

### 3.2. Sensitivity to Including Partly Cloudy Conditions

The cloud fraction is derived by computing the cloud cover as identified by CALIPSO over a given grid cell; therefore, there is more noise in computing the cloud fraction when the sky cover is partly cloudy as opposed to completely clear or cloudy. Thus, we tested providing the model training data with only the clear or completely cloudy conditions (i.e., cloud fraction equals 0 or 1) versus providing the model training data composed of all cloud conditions including partly cloudy (i.e., cloud fraction in the range from 0 to 1). We found when the RF was trained on all conditions and applied on all conditions the validation dataset accuracy was 91.7% using the best threshold as described in Section 3.3. Here, accuracy is defined as the percentage of correctly predicted clear and cloudy conditions divided by the total number of predictions. When we trained the model on only the instances that were clear or cloudy and applied to all conditions, the accuracy decreased to 89.5%, which indicates that including the partly cloudy conditions improved the model. The mean absolute error (MAE) of the RF trained on all instances (including partly cloudy) or just predicting the cloud fraction was 0.135. Therefore, our final model was trained on all instances, which likely improved the error by allowing the RF to better learn the predictive relationships even in partly cloudy conditions. Ultimately, we need to predict binary cloud fraction or cloud mask for initialization of NWP models; hence, we do not utilize the specific cloud fraction values other than to determine thresholds for binary cloud fraction.

### 3.3. Optimal Random Forest Configuration

**Figure 6.**
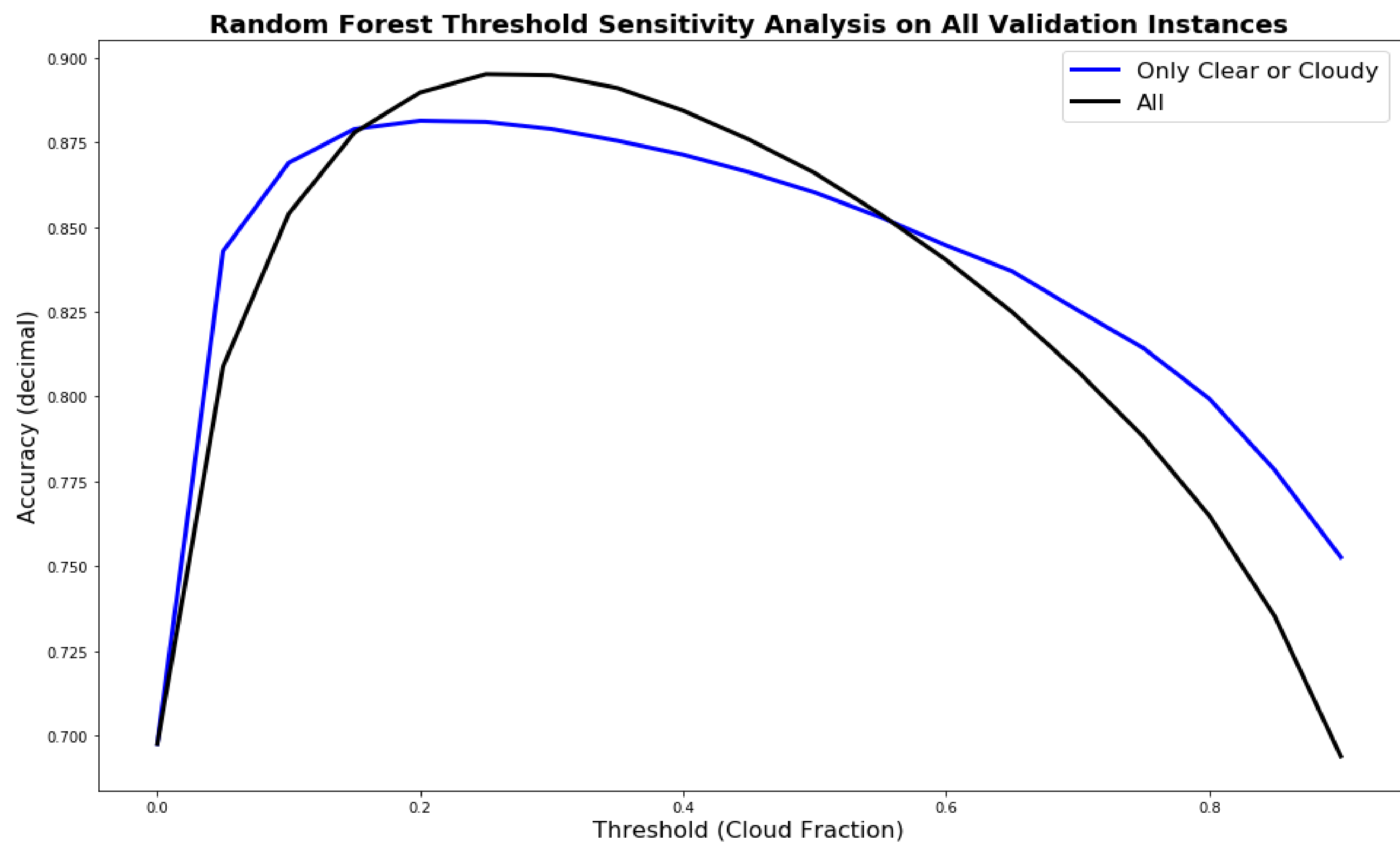
Sensitivity study for determining the optimal threshold for determining clear (0) versus cloudy (1) with training on either all instances or just the clear or completely cloudy instances. The optimal threshold was determined to be 0.25 for the training dataset with all instances.

As part of other ongoing projects in developing a RF parameterization in the WRF model, we were limited to the complexity of the RF configuration. Specifically, we were limited to using a RF regression model rather than a RF classifier, and we were limited to having a maximum of 200 trees in the forest. The later limitation is relatively minor as 200 trees was satisfactory for this prediction problem. The former limitation actually provides more flexibility as we were able to perform a sensitivity study to determine the optimal threshold. We evaluated the optimal threshold based on the accuracy score for both providing the model that consisted of all cloud conditions or just the clear or cloudy training instances. The results are shown in Figure 6 where the accuracy for all cloud instances is shown as a black line and the only clear or cloudy instances is shown as a blue line. The optimal threshold choice was determined to be 0.25 with values greater than 0.25 being set equal to 1 (cloudy) and less than or equal to 0.25 set equal to 0 (clear).

Finally, we computed the confusion matrix for the final configuration of the RF on the test dataset held out for independent verification. The confusion matrix is shown in Table 1. The overall accuracy on the test dataset is 86.2%, which is slightly lower than the validation dataset used in determining the optimal configuration. The RF model tends to overestimate cloudy conditions as the accuracy is higher for cloudy (93.4%) than for clear conditions (67.5%), which is logical given there are more cloudy instances than clear instances in the test dataset.

**Figure 7.**
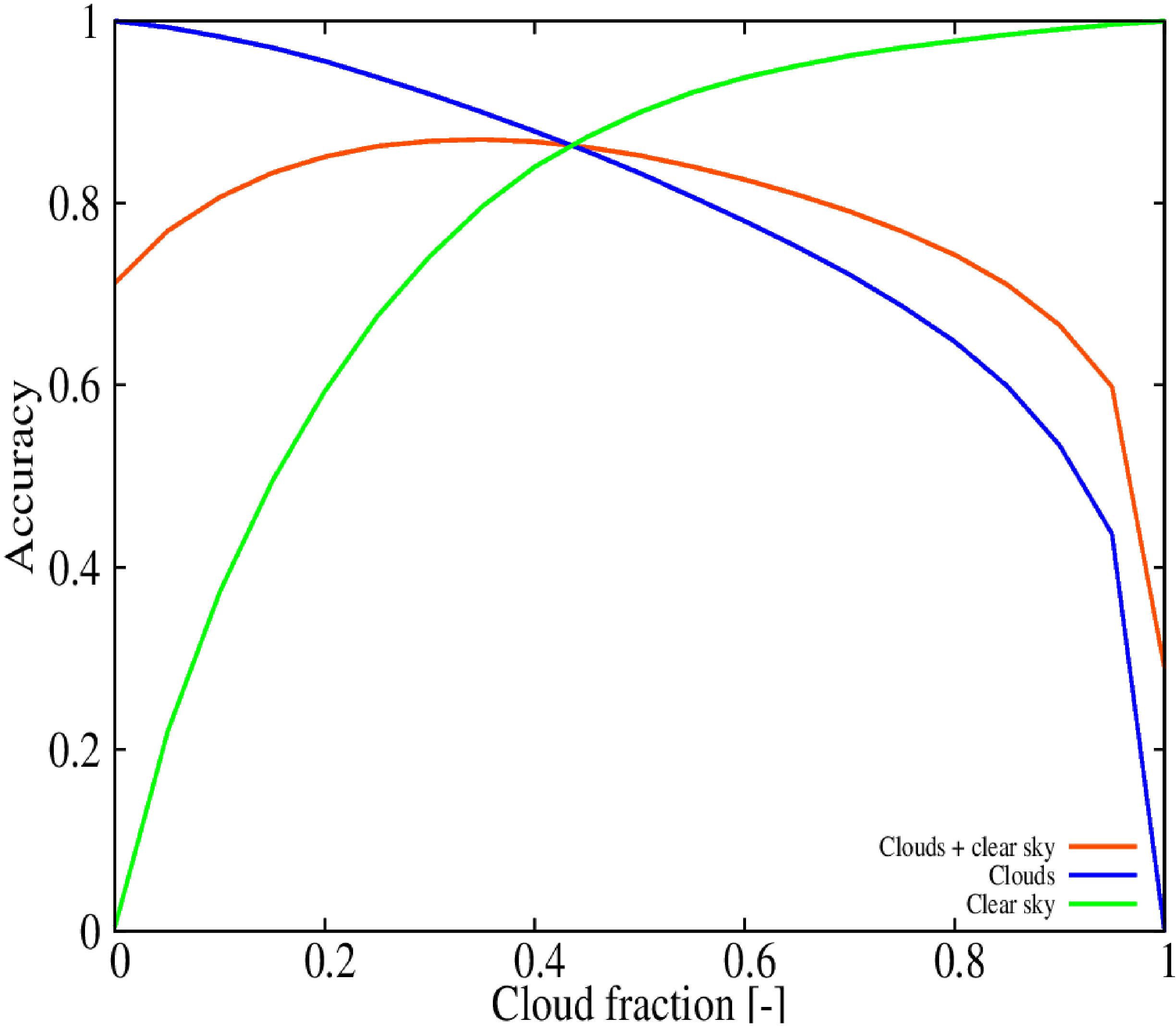
Accuracy of the optimal RF configuration calculated with the testing dataset.

The performance of the RF can be understood after analyzing the accuracy as a function of the cloud fraction threshold for the testing dataset (Figure 7). The threshold selected with the validation dataset, 0.25, is near the maximum of the overall accuracy function (red line). However, the maximum is smooth, and increasing the threshold to 0.4 shows a slightly higher overall accuracy with an increase of the clear sky detection (green line) at a cost of reducing the performance of the cloud detection (blue line). These improvements are summarized in the confusion matrix shown in Table 2. The accuracy is higher for the cloud detection (87.8%) than for the clear sky (83.9%), but their values are more similar.

### 3.4. Comparsion to Clear Sky Mask Product from GOES-16

In order to better evaluate the performance of the RF model, we have compared our results to the operational GOES-16 level 2 clear sky mask product. This product is generated from the ABI retrievals at 2-km resolution (Algorithm Theoretical Basis available at https://www.star.nesdis.noaa.gov/goesr/documents/ATBDs/Baseline/ATBD_GOES-R_Cloud_Mask_v3.0_July%202012.pdf). Data from the same temporal period covered by the testing dataset was spatio-temporally matched to the CALIPSO cloud mask in a similar way as the GOES-16 radiances (see Section 2.1). By so doing, the product is interpolated from the original 2 km resolution to a coarser grid, 9 km, which allows us to have a cloud fraction in our target grid. Only retrievals flagged as good quality or degraded due to large local zenith angle were used. The use of retrievals degraded due to large local zenith angles is necessary to have data over the complete 9-km grid. The cloud mask is calculated in a similar way as with CALIPSO retrievals by defining clear sky as those grid points with zero cloud fraction and cloudy otherwise. The confusion matrix is shown in Table 3. The GOES-16 product shows an overall accuracy of 85.7% with a cloud detection accuracy of 93.0% and a clear sky detection of 67.7%. These numbers are similar to the ones we obtained with our RF algorithm depending on our choice of threshold for clear vs cloudy.

**Figure 8.**
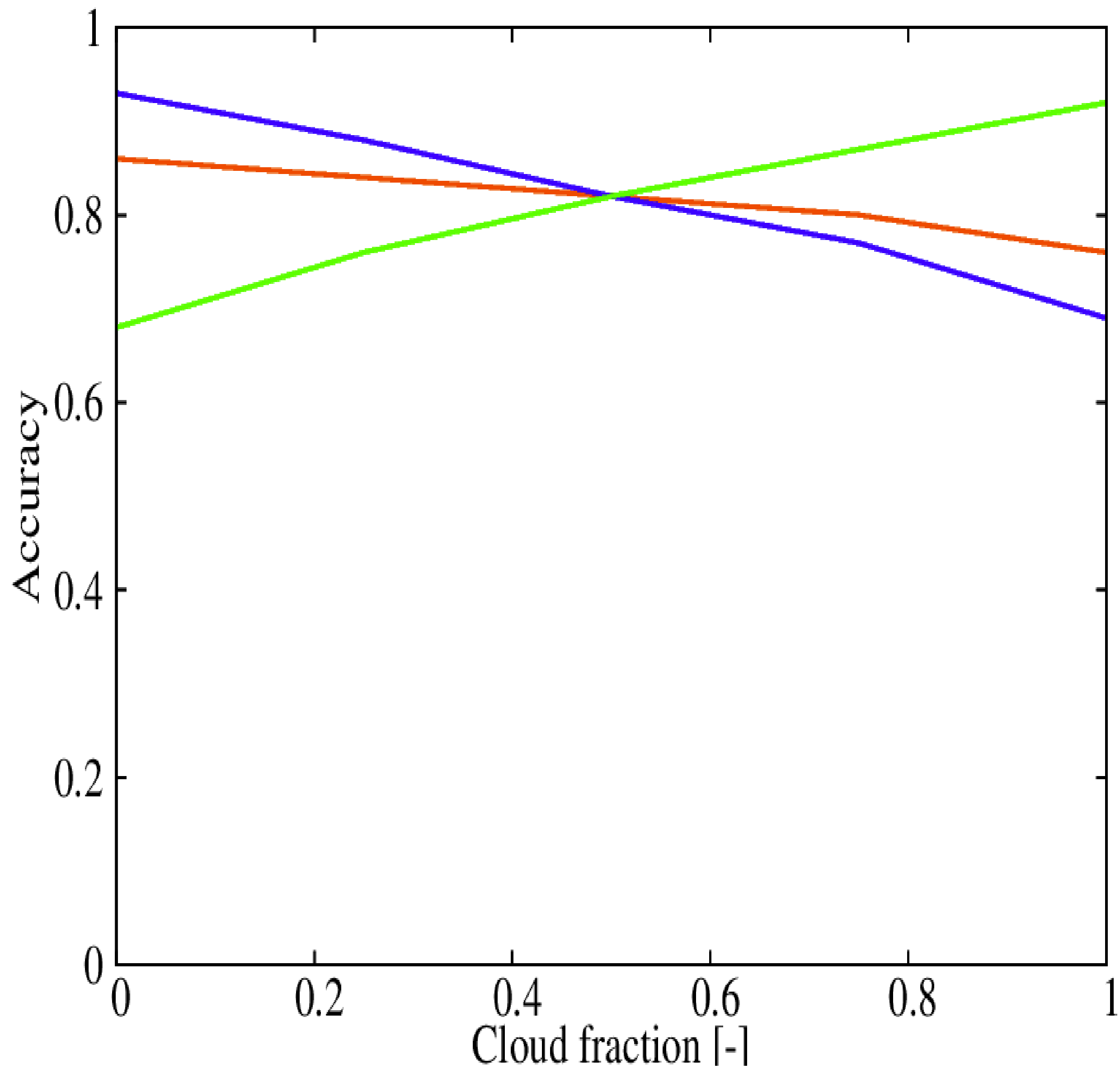
Accuracy of the optimal GOES-16 clear sky product as a function of a cloud fraction threshold.

Although there is not a theoretical basis supporting an increase in the cloud fraction threshold selected to define the cloud mask calculated with the GOES-16 clear sky product, we have inspected potential sensitivities. In particular, we have calculated the accuracy for four additional thresholds: 0.25, 0.50, 0.75 and 1.00. These values together with the previous one using the 0.0 threshold are shown in Figure 8. Results show resemblances with the calibration of the RF model (Figure 7). At a cloud fraction of 0.50, the performance of the cloud and the clear sky detections are the same. The overall accuracy is 82.2%, and the same value is obtained for the cloud detection accuracy and the clear sky accuracy. This result may suggest a tendency to over-predict the cloud coverage by the GOES-16 product.

**Figure 9.**
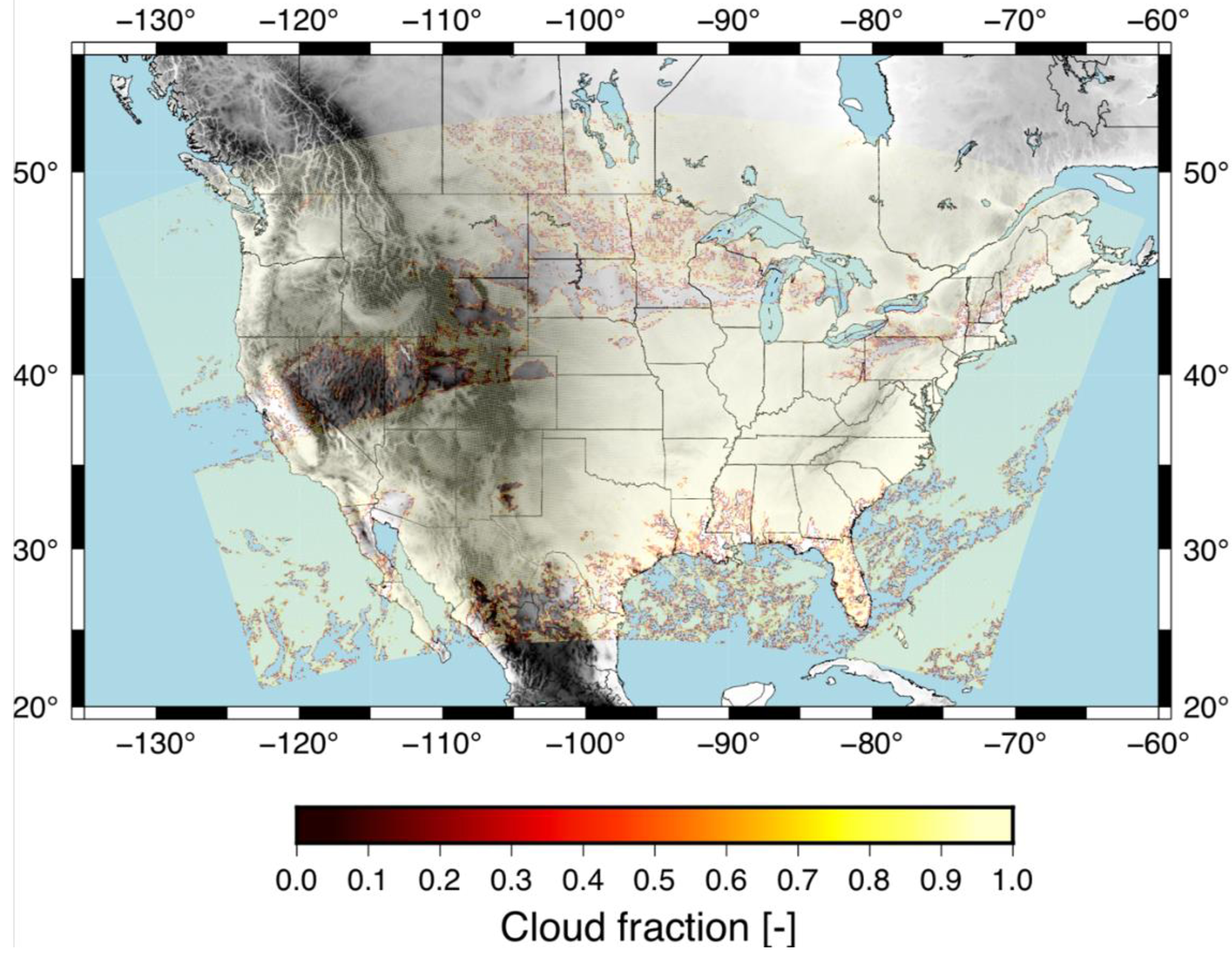
GOES algorithm cloud mask product for 1 April 2018 at 19Z.

**Figure 10.**
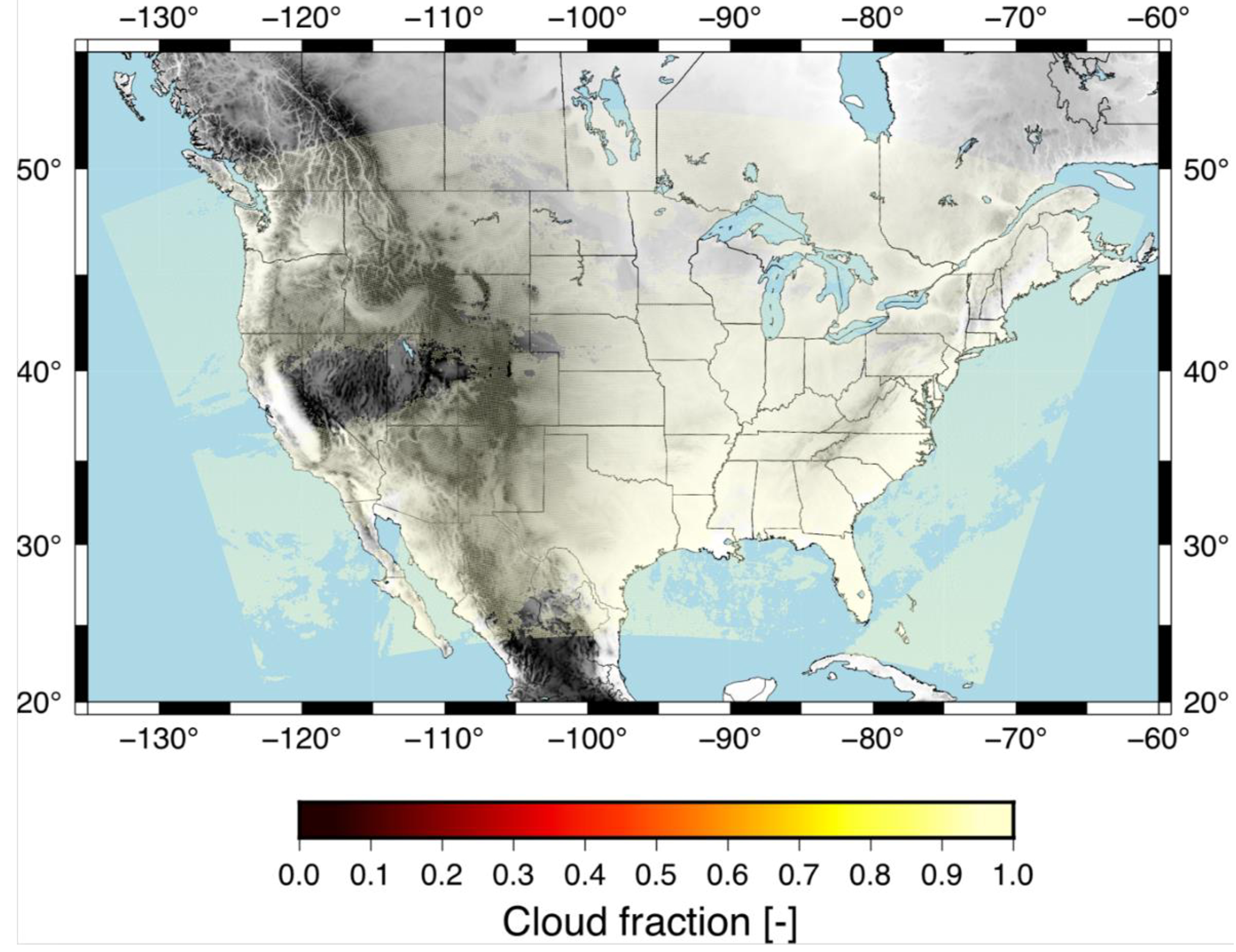
RF algorithm cloud mask product for 1 April 2018 at 19Z.

We have evaluated the RF based cloud mask compared to GOES algorithm for 1 April 2018 at 19Z, which shows similar cloud mask for the two algorithms. We present the results for the GOES algorithm in Figure 9 and the RF based algorithm in Figure 10. Here, you can see that both models generally capture the clouds over the same areas (yellow) with clear skies indicated with no color. There are small differences such as the GOES algorithm has slightly less clouds in the mountains, such as in Wyoming and southern Montana compared to the RF based cloud mask. The RF also tends to have more continuous clear skies over Minnesota (north-central US) compared to the GOES algorithm that hdtas more intermittent cloud cover.

## 4. Discussion

Improving the accuracy of cloud detection has significant potential applications in short-term weather forecasting using NWP models. In this study we have focused on improving cloud detection prediction using a machine learning method to capture the non-linear relationships among GOES-R remote sensing data with cloud detection. An intermediate step in the cloud detection retrieval is the retrieval of the cloud fraction on our target grid, which can be used to complement other GOES-R cloud products and thus better characterize the initial cloud field. More accurate cloud detection could have significant improvements in short-term solar power prediction that would allow for better utilization of solar power in the energy grid and potentially better optimization of ancillary services or co-optimization with battery storage systems.

**Figure 11.**
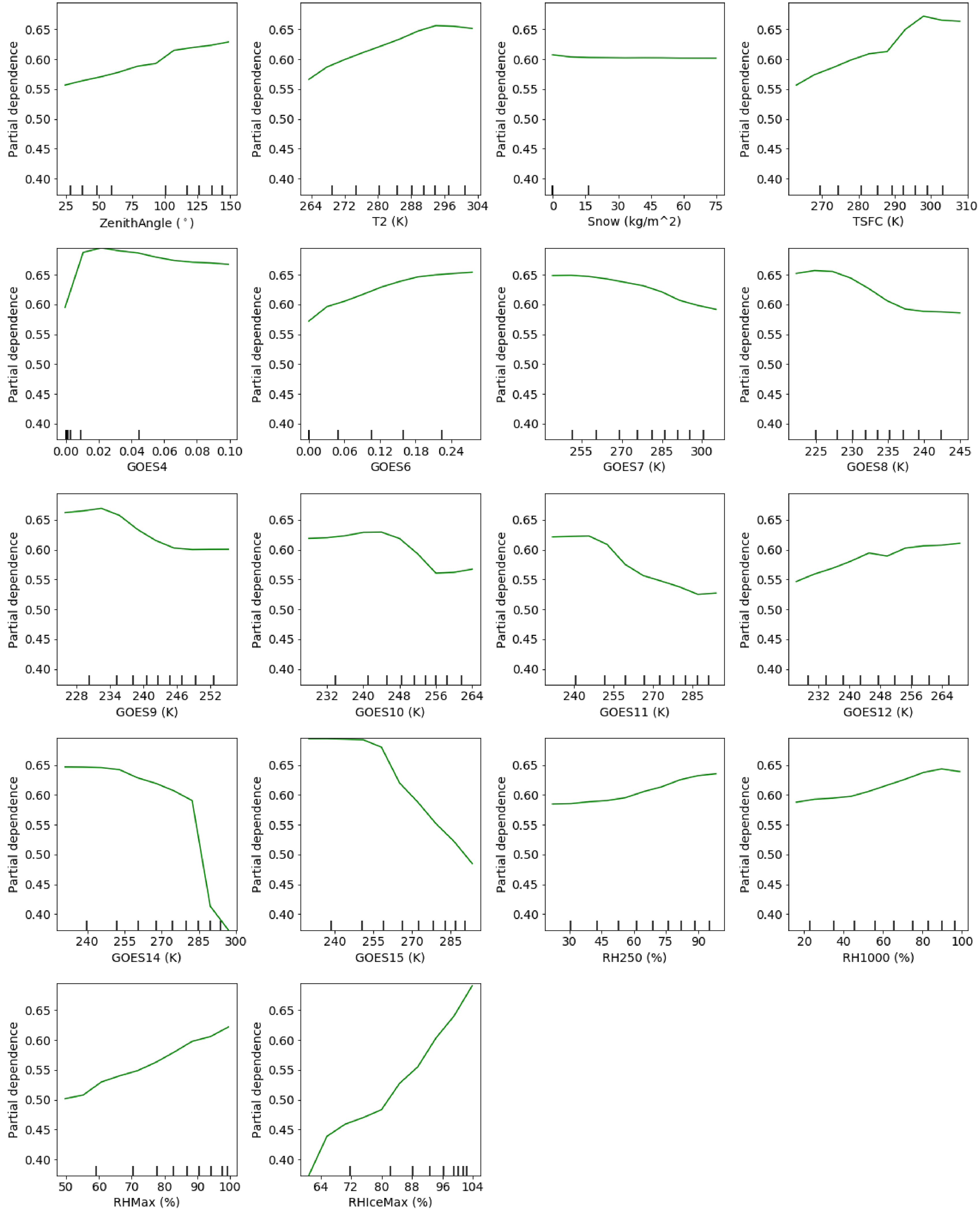
Predictor importance plots showing the relative change in prediction based on each of the variables used in the final model.

One of the benefits of the RF model is the additional interpretability using tools such as partial dependence plots. We examined the partial dependence of the cloud fraction prediction based upon each of the variables as shown in Figure 11. Here we can see that some variables have linear relationships with the cloud fraction prediction, such as RHMax and RHIceMax while others have non-linear relationships, such as GOES channel 4, GOES channel 14 and GOES channel 15. There is minimal change in the partial dependence for snow, but since it is low on the predictor importance, the dependence of the change of that variable has little impact in the final predictions. As shown in Figure 4, the top 5 predictors in terms of predictor importance of RHIceMax, GOES channel 15, GOES channel 4, T2 and TSFC (2 m and surface temperature). The partial dependence of the relative humidity for ice-based clouds increases linearly with an increase in relative humidity. This makes physical sense as a higher maximum relative humidity would lead to a greater chance of cloud formation when the actual observed relative humidity reaches 100% (i.e., saturation). For GOES channel 15 and GOES channel 4, there are clearly values at which the relationship starts linearly decreasing. Specifically, GOES channel 15 brightness temperature values near 255 indicate sharp transition in the cloud fraction prediction and highlight the value of the RF to capture the regime change. The temperatures at the surface and 2 m see increasing cloud fraction when the temperature increases, which may indicate higher chance of clouds due to strong convection as temperatures increases (i.e., more atmospheric instability). This analysis somewhat complements the analysis by [27] that described the performance and major biases of the ABI Cloud Height Algorithm (ACHA), used by GOES-R satellites, to retrieve the cloud top height and cloud top temperature over the Southern Ocean using Himawari-8 retrievals.

The performance of the RF cloud detection is comparable with the GOES-16 ACM product. The overall accuracy of the RF model is 86.2% whereas for the ACM product is 85.7%. The cloud/clear sky accuracy is also similar for both products with values of 93.4/67.5 for the RF model and 93.0/67.7 for the ACM product.

Potential improvements of the RF algorithm are being examined at the time of writing. We are exploring adding additional meteorological variables as predictors that can further increase the performance of the cloud mask predictions. In addition, we are in the process of filling in some gaps in NCAR’s archives of both GOES-16 and HRRR datasets to enlarge the training and validation datasets. Another aspect under investigation is the potential benefit of refining the spatio-temporal matching of the predictand and predictor datasets. For example, CALIPSO retrievals were assigned to the nearest hour to maximize the size of the predictand dataset. By refining this temporal criteria, we should have a clearer signal between the predictand and predictor datasets but at the cost of reduced datasets, and the impact of this is not clear. In this direction, we are also inspecting sensitivities to the available CALIPSO retrievals per grid cell. In this study, we calculated the cloud fraction if at least two CALIPSO retrievals were available in a given cell. Enlarging the minimum number of retrievals should contribute to more statistically robust conclusions. This also may affect the validation of the GOES-16 clear sky product and further evaluation is underway.

The RF algorithm has been already implemented inside of MAD-WRF. With this aim, we are ingesting GOES-16 radiances which are combined with the other predictor variables to create the cloud mask at initialization time. The cloud mask is combined with cloud top height estimations (e.g., the ACHA level 2 product) and cloud base height observations from METAR stations to have a complete characterization of the three-dimensional cloud field. This information is combined with the hydrometeor content at initial time, if available, and an adiabatic cloud model to initialize the hydrometeor content of the clouds. Having this cloud initialization in place is allowing us to inspect the added value imposing the cloud mask has on the solar irradiance predictions. The irradiance is very sensitive to the cloud mask, and the on-going evaluations already point out the positive impact of imposing an accurate cloud mask. In this direction, we have shown that the RF algorithm for cloud mask prediction is a very promising machine learning algorithm to improve the state-of-the-science for cloud detection from satellite data with a potential to greatly improve short-term solar power forecasts based on NWP models such as MAD-WRF.

## Supplementary Material

dataset1_training_dataset

dataset2_testing_dataset

dataset3_data_for_fig10

## Figures and Tables

**Table 1.** Confusion matrix for optimal RF configuration on the independent test dataset.

Confusion Matrix	Actual Clear	Actual Cloudy
Predicted Clear	65,585 (67.5%)	14,720 (7.1%)
Predicted Cloudy	31,474 (32.4%)	224,105 (93.4%)

**Table 2.** Confusion matrix for optimal RF configuration on the independent test dataset and a cloud fraction threshold of 0.40.

Confusion Matrix	Actual Clear	Actual Cloudy
Predicted Clear	83.9%	12.2%
Predicted Cloudy	16.1%	87.8%

**Table 3.** Confusion matrix for the GOES-16 Clear Sky Mask product.

Confusion Matrix	Actual Clear	Actual Cloudy
Predicted Clear	67.7%	7.0%
Predicted Cloudy	32.3%	93.0%
